# Transient peripheral vestibular hypofunction measured with vestibular short-latency evoked potentials following noise exposure in rats

**DOI:** 10.1152/jn.00131.2021

**Published:** 2021-09-22

**Authors:** Courtney E. Stewart, David S. Bauer, Richard A. Altschuler, William Michael King

**Affiliations:** ^1^ VA Ann Arbor Healthcare System, Ann Arbor, Michigan; ^2^Department of Otolaryngology/Head-Neck Surgery, Kresge Hearing Research Institute, University of Michigan, Ann Arbor, Michigan

**Keywords:** noise-induced hearing loss, noise-induced peripheral vestibular hypofunction, temporary threshold shift, vestibular, VsEP

## Abstract

Exposure to 120 dB sound pressure level (SPL) band-limited noise results in delayed onset latency and reduced vestibular short-latency evoked potential (VsEP) responses. These changes are still present 4 wk after noise overstimulation. Noise-induced hearing loss (NIHL) has been shown to vary in extent and duration based on the noise intensity. This study investigated whether noise-induced peripheral vestibular hypofunction (NPVH) would also decrease in extent and/or duration with less intense noise exposure. In the present study, rats were exposed to a less intense noise (110 dB SPL) but for the same duration (6 h) and frequency range (500–4,000 Hz) as used in previous studies. The VsEP was assessed 1, 3, 7, 14, 21, and 28 days after noise exposure. In contrast to 120 dB SPL noise exposure, the 110 dB SPL noise exposures produced smaller deficits in VsEP responses that fully recovered in 62% (13/21) of animals within 1 wk. These findings suggest that NPVH, a loss or attenuation of VsEP responses with a requirement for elevated stimulus intensity to elicit measurable responses, is similar to NIHL, that is, lower sound levels produce a smaller or transient deficit. These results show that it will be important to determine the extent and duration of vestibular hypofunction for different noise exposure conditions and their impact on balance.

**NEW & NOTEWORTHY** This is the first study to show a temporary noise-induced peripheral vestibular hypofunction that recovers following exposure to continuous noise.

## Graphical abstract

**Figure:**
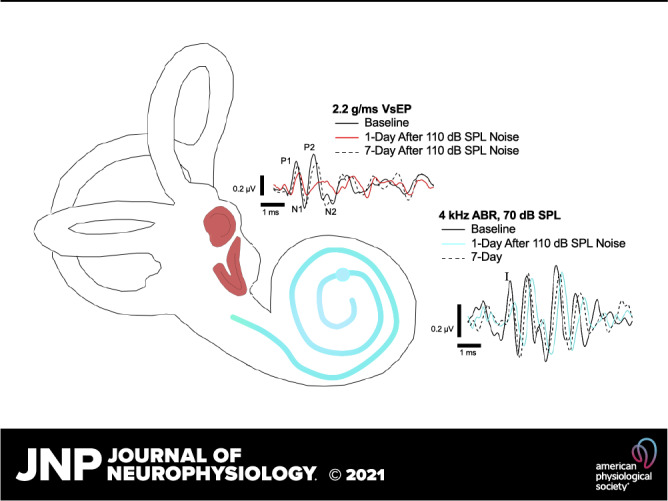

## INTRODUCTION

Multiple characteristics of noise exposure, for example, frequency bandwidth, sound level, exposure duration, and time course, are likely to determine the extent of peripheral damage (for review, see Ref. 1). Our previously published data (2) show that intense noise exposure can cause a vestibular deficit characterized by reductions in peripheral and central vestibular responses to linear head-jerk stimuli. Specifically, intense noise exposure causes attenuated and delayed vestibular short-latency evoked potential (VsEP) responses to head jerks, indicating peripheral vestibular hypofunction. Peripheral vestibular hypofunction or loss was still present 28 days after the noise exposure depending on stimulus intensity. Of particular importance is whether noise-induced peripheral vestibular hypofunction (NPVH) would be less or transient after a less intense noise exposure condition.

Prior studies have compared the effects of noise on vestibular pathology and balance by varying noise frequency (3), exposure duration (4), and time course (5). Akdogan et al. (5) identified damage to guinea pig vestibular sensory epithelia exposed to continuous but not intermittent noise; however, the recovery from the noise exposures was not examined. Tamura et al. (3) reported functional motor deficits related to vestibular loss in mice for 4 wk after 1-mo continuous 70 dB sound pressure level (SPL) low- but not high-frequency noise exposure. Mice chronically exposed to low-frequency noise had impaired rotarod performance, more frequent balance beam crossing failures and reduced stride length. Hsu et al. (4) reported recovery of vestibular evoked myogenic potential (VEMP) responses and auditory brainstem response (ABR) thresholds in guinea pigs after brief (30 min) noise exposure (115 dB SPL broadband noise). Furthermore, they reported normal saccular morphology in guinea pigs with recovery of both VEMP and ABR. Taken together, these studies suggest variable recovery of auditory and vestibular function dependent on noise exposure conditions. However, Hsu et al. did not examine the effects of sound levels of equal duration and bandwidth and did not use any longitudinal measure (e.g., VsEP) to assess noise-induced vestibular injury and recovery. We reported that a 6-h exposure to 120 dB SPL 1.5-kHz 3-octave band noise (OBN) produced a reduction in VsEP amplitude and prolonged VsEP latency that was still present 28 days after noise exposure (2). This frequency range activates the most apical 20% of the cochlea and was, therefore, selected as an adequate low-frequency range for our rat model (6). In addition, it is well-recognized that low-frequency sound within the range selected for this study and prior work (2) is most activating to mammalian otolith organs (for review, see Ref. 7) and is most effective in damaging the otolith organs (3). Therefore, the present study used the same noise exposure duration and bandwidth (6-h, 1.5-kHz 3-OBN) but reduced intensity to 110 dB SPL to determine whether there would be less effect on VsEP responses and partial or full recovery following exposure to the lower sound level.

Our previous study reported that 120 dB SPL noise exposure produced not only peripheral vestibular hypofunction but also auditory brainstem response (ABR) threshold shifts (TS) that persisted 28 days after noise. If noise exposure duration and intensity are increased, noise-induced ABR TS can change from temporary TS (TTS) to permanent TS (PTS). Therefore, the present study also assessed ABR TS and wave I characteristics at the lower sound level.

## METHODS

### Animals

Long–Evans rats initially weighing 350–400 g (Charles River Laboratories) were housed in pairs on a 12:12-h light-dark cycle (lights on at 0800 and off at 2000) with ad libitum access to food and water. Following baseline ABR and VsEP measurements (*n* = 15 and *n* = 21, respectively, unless otherwise specified), rats were exposed to 110 dB SPL 1.5-kHz-centered 3-OBN for 6-h and then retested at 1, 3, 7, 14, 21, and 28 days post noise exposure (VsEP) and 1, 3, 7, and 28 days postnoise exposure (ABR). All procedures were carried out in accordance with National Institutes of Health guidelines and were approved by the Institutional Animal Care and Use Committee at the University of Michigan.

### Surgical Preparation

Rats’ heads were positioned on a stereotaxic frame under isoflurane anesthesia and a dorsal cranial midline incision was made to expose bregma and lambda. The skull was leveled with respect to the stereotaxic frame by adjusting the pitch of the head until the *Z*-axis coordinate of bregma and lambda was equal. After the skull was leveled, two anchor screws were placed into the skull and a custom head bolt was bonded to the skull at bregma with C&B Metabond cement (Parkell, Inc., Edgewood, NY). After the cement was set, the head bolt was fused to the anchor screws in the skull with dental acrylic and the rats were allowed to recover for 10 days.

### Noise Exposure Parameters

The continuous free-field noise stimulus (1.5-kHz 3-OBN) was designed to impact the most apical 20% of the rat cochlea or the lower end of the rat-hearing frequency range (6, 8, 9). The noise was delivered at a maximum intensity of 110 dB SPL, which is comparable in intensity with a loud rock concert or sporting event. Unanesthetized Long–Evans rats were placed into individual wire mesh cages and held in a ventilated sound exposure booth for 6 h. Details of the free-field noise exposure paradigm used in this study have been described previously (2). After noise exposure, the rats were returned to their home cages to recover.

### ABR Protocol

The first wave of the ABR is produced by the synchronous firing of auditory nerve fibers arising from spiral ganglion cells and has been used as an indicator of cochlear synaptopathy following noise exposure (10). In this study, the ABR was measured at 8, 4, and 1.5 kHz (*n* = 12, unless otherwise noted) as described previously (2). Briefly, needle electrodes were placed at the vertex (active), below the test ear (reference), and in the hip (ground) and pure tone stimuli of alternating polarity were delivered through a speculum placed just inside the ear canal next to the tragus. Stimuli were presented in 15-ms-duration tone bursts with 1-ms rise and fall times, at a rate of 10/s. Responses (1,024) were averaged for each stimulus intensity, and this procedure was repeated at each frequency. Response waveforms were collected for stimulus levels in 10-dB steps at higher stimulus levels with additional 5-dB steps near threshold. Thresholds were interpolated between the lowest stimulus level where a response was observed and 5-dB lower where no response was observed. ABR wave I amplitudes were determined as the amplitude of the first positive peak minus the amplitude of the negative valley. ABR wave I latency values were determined as the time at which the P1 peak occurred relative to the stimulus peak.

### VsEP Protocol

**Figure 1.**
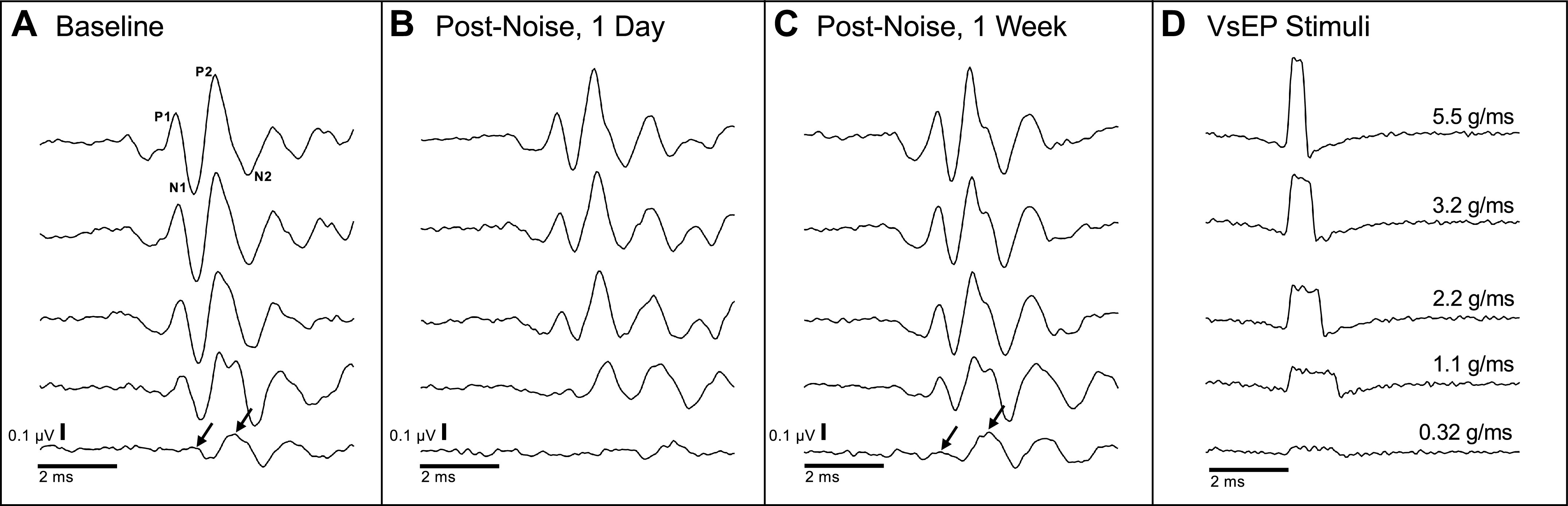
Vestibular short-latency evoked potential (VsEP) response recovery from a representative rat. VsEP responses have two distinct peaks (P1, P2) and troughs (N1, N2; *A*, *top* traces). P1N1 is related to vestibular nerve activity, and P2N2 is related to activity in the vestibular nuclear complex. Responses to head-jerk stimuli ranging from 0.32 to 5.5 g/ms (*D*, *bottom* to *top*) are shown for a representative rat exposed to 110 dB sound pressure level (SPL) noise. At baseline, small, yet distinct, P1N1 and P2N2 waveforms were visible in response to the smallest head-jerk stimulus (*A*, *bottom* trace, arrows). One day after noise exposure, no discernable response could be elicited, suggesting a threshold shift (*B*, *bottom* trace). One week after noise exposure, this response recovered (*C*, arrows).

The VsEP is a far-field electrical potential produced by action potentials in the vestibular portion of the eighth nerve (11–14). The VsEP selectively samples large-diameter irregular afferent fibers that are known to fire synchronously in response to auditory stimulation and rapid head jerks (15–17). The VsEP was measured longitudinally in 110 dB SPL noise-exposed rats (*n* = 21, unless otherwise noted) according to the method established by Jones et al. (17) and described previously by Stewart et al. (2). Briefly, rats’ heads were fixed to a shaker and stimuli of constant peak acceleration (iso-acceleration) ranging from ∼0.32 g/ms to 5.5 g/ms were delivered in the naso-occipital direction. Electrodes (stainless steel needles) placed subcutaneously at the vertex (noninverting), mastoid (reference), and hip (ground) recorded the VsEP. Stimuli were delivered as successive positive and negative head jerks (200 of each) and averaged together to minimize electromagnetic artifacts from the shaker. Figure 1 illustrates typical averaged VsEP recordings obtained in our laboratory with these stimuli.

Signals from the electrodes were amplified (×200,000), filtered (300–10,000 Hz), and digitized by a CED data acquisition system (Cambridge Electronic Design, Cambridge, UK) at 20 kHz and analyzed using custom software (MATLAB); the average was synchronized using the stimulus onset as a trigger. VsEP waveform amplitude values were determined as the amplitude of the first positive peak (P1) minus the amplitude of the negative (N1) valley. VsEP waveform latency values were determined as the time at which the P1 peak occurred relative to the stimulus peak (Fig. 1). VsEP thresholds were determined as the lowest level at which a custom MATLAB script designed to detect VsEP peaks and valleys could reliably identify waveforms and/or the lowest response that could be visually identified. By including visual identification in addition to objective MATLAB analysis, thresholds could be confirmed, especially when responses were present but severely attenuated. Data from noise-exposed rats (110 dB SPL, *n* = 21, unless otherwise noted below) were grouped by day and compared against the baseline data with repeated-measures ANOVA in the same animals at 1, 3, 7, 14, 21 (*n* = 20), and 28 (*n* = 19) days after noise exposure.

## RESULTS

### VsEP

Peripheral vestibular hypofunction was characterized by threshold shifts in the stimulus intensity required to elicit measurable VsEP responses, reduced VsEP amplitudes, and increased VsEP latencies. In most animals (13/21), the effects of noise significantly recovered within the first week after noise exposure. Twenty-eight days after noise exposure, two additional rats (total, 15/19) had VsEP thresholds that returned to baseline, indicating further recovery after the first postnoise week.

### VsEP Threshold Shift

**Figure 2.**
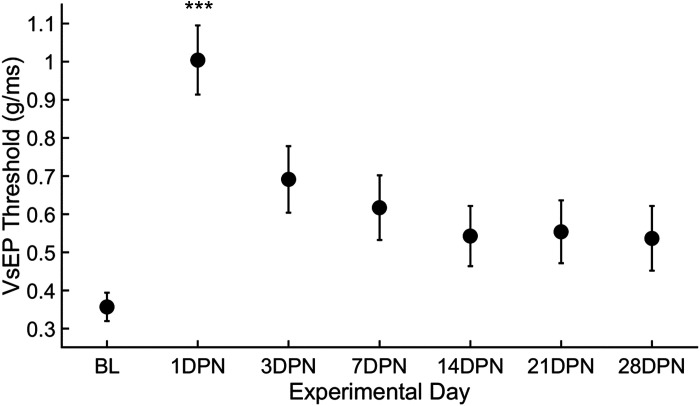
Vestibular short-latency evoked potential (VsEP) threshold shifts recovered over the first postnoise exposure week and then stabilized. VsEP thresholds were significantly elevated from baseline (*n* = 21) 1 day after noise exposure (*n* = 21). Although thresholds appeared elevated from baseline thresholds 3 and 7 days after noise exposure (*n* = 21), they were not significantly different from baseline, and this recovery was stable 14 (*n* = 21), 21 (*n* = 20), and 28 (*n* = 19) days after noise exposure (error bars = SE, ***P < 0.001). BL, baseline; DPN, days postnoise; *n*, number of rats.

Although there was no main effect of measurement day (*F*_6,102_ = 0.36339, *P* = 0.90; repeated-measures ANOVA), VsEP thresholds increased 1 day after noise exposure (Fig. 2; *P* < 0.001, Dunn’s multiple-comparisons test). Three days after the noise exposure, VsEP thresholds were not statistically different from baseline but appeared elevated and remained this way at subsequent timepoints.

### VsEP Amplitude

#### P1N1 waveform amplitude.

**Figure 3.**
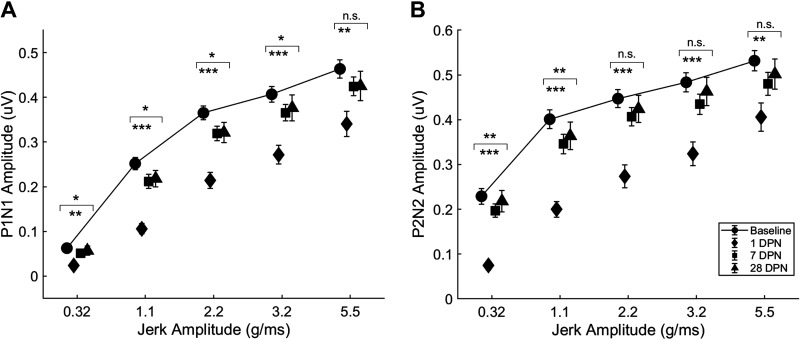
Vestibular short-latency evoked potential (VsEP) waveform amplitude was temporarily reduced following exposure to 110 dB sound pressure level (SPL) continuous noise. *A*: P1N1 amplitude was significantly reduced in response to 0.32, 1.1, 2.2, 3.2, and 5.5 g/ms head-jerk stimuli 1 day after noise exposure (diamonds) vs. baseline (circles, solid line). Amplitudes were similar to baseline at subsequent timepoints. *B*: P2N2 waveform amplitude was significantly reduced in response to 0.32, 1.1, 2.2, 3.2, and 5.5 g/ms head-jerk stimuli 1 day after noise exposure (diamonds) vs. baseline (circles, solid line). Amplitudes were similar to baseline at subsequent timepoints (baseline, 1, 7 days postnoise, *n* = 21; 28 days postnoise, *n* = 19; error bars = SE; *P < 0.05; **P < 0.01; ***P < 0.001, asterisks above brackets = group effect identified with ANOVA, asterisks below brackets = significant findings 1 day after noise exposure identified with Dunn’s multiple-comparisons test). DPN, days postnoise; *n*, number of rats; n.s., not significant.

Significant reductions in VsEP P1N1 amplitude occurred in response to 0.32 (*F*_6,102 _= 2.4157; *P* < 0.05), 1.1 (*F*_6,102_ = 2.8180; *P* < 0.05), 2.2 (*F*_6,102_ = 2.5516; *P* < 0.05), and 3.2 g/ms stimuli (*F*_6,102 _= 2.4081; *P* < 0.05) but not in response to the 5.5 g/ms stimulus (*F*_6,102 _= 1.0436, *P* = 0.4018; repeated-measures ANOVA). One day after noise exposure, VsEP P1N1 amplitude was significantly reduced in response to 0.32 (*P* < 0.01), 1.1(*P* < 0.001), 2.2 (*P* < 0.001), 3.2 (*P* < 0.001), and 5.5 g/ms (*P* < 0.01; Dunn’s multiple-comparisons test). Subsequently, VsEP P1N1 amplitudes returned to baseline (Fig. 3*A*).

#### P2N2 waveform amplitude.

Significant reductions in VsEP P2N2 amplitude occurred in response to 0.32 g/ms (*F*_6,102_ = 3.1029; *P* < 0.01) and 1.1 g/ms stimuli (*F*_6,102_ = 3.2107; *P* < 0.01) but not in response to 2.2 g/ms (*F*_6,102_ = 0.7635, *P* = 0.6003), 3.2 g/ms (*F*_6,102 _= 1.1411, *P* = 0.3441), or 5.5 g/ms stimuli (*F*_6,102_ = 0.7674, *P* = 0.5972; repeated-measures ANOVA). One day after noise exposure, VsEP P2N2 amplitude was significantly reduced in response to 0.32 g/ms, 1.1 g/ms, 2.2 g/ms, 3.2 g/ms (*P* < 0.001), and 5.5 g/ms stimuli (*P* < 0.01; Dunn’s multiple-comparisons test). Subsequently, VsEP P2N2 amplitudes returned to baseline (Fig. 3*B*).

### VsEP Latency

#### P1 latency.

**Figure 4.**
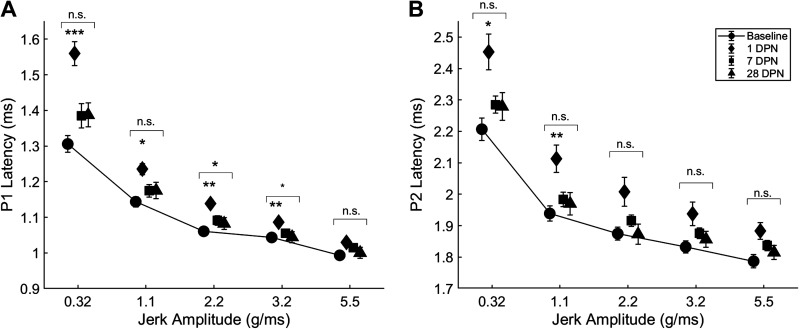
Vestibular short-latency evoked potential (VsEP) peak latency was temporarily prolonged following exposure to 110 dB sound pressure level (SPL) continuous noise. *A*: P1 latency was significantly prolonged in response to 0.32, 1.1, 2.2, and 3.2 g/ms head-jerk stimuli 1 day after noise exposure (diamonds) vs. baseline (circles, solid line); latencies were similar to baseline at subsequent timepoints. *B*: P2 latency was significantly prolonged in response to 0.32 and 1.1 g/ms head-jerk stimuli 1 day after noise exposure (diamonds) vs. baseline (circles, solid line); latencies were similar to baseline at subsequent timepoints (baseline, 1, 7 days postnoise, *n* = 21; 28 days postnoise, *n* = 19; error bars = SE; *P < 0.05; **P < 0.01; ***P < 0.001, asterisks above brackets = group effect identified with repeated-measures ANOVA, asterisks below brackets = significant findings 1 day after noise exposure identified with Dunn’s multiple-comparisons test). DPN, days postnoise; *n*, number of rats; n.s., not significant.

VsEP P1 latency was significantly prolonged in response to 2.2 g/ms (*F*_6,102_ = 2.8157, *P* < 0.05) and 3.2 g/ms stimuli (*F*_6,102_ = 2.2326, *P* < 0.05) but not in response to 0.32 g/ms (*F*_6,102_ = 1.1299, *P* = 0.3504), 1.1 g/ms (*F*_6,102_ = 1.6034, *P* = 0.1538), or 5.5 g/ms stimuli (*F*_6,102_ = 1.2979, *P* = 0.2649; repeated-measures ANOVA). VsEP P1 latency was significantly prolonged 1 day after noise exposure in response to 0.32 g/ms (*P* < 0.001), 1.1 g/ms (*P* < 0.05), 2.2 g/ms (*P* < 0.01), and 3.2 g/ms (*P* < 0.01; Dunn’s multiple-comparisons test). Subsequently, VsEP P1 latencies returned to baseline (Fig. 4*A*).

#### P2 latency.

Although there was no main effect of noise exposure on VsEP P2 latency in response to any stimulus (0.32, *F*_6,102_ = 0.7270, *P* = 0.6289; 1.1, *F*_6,102 _= 1.3187, *P* = 0.2556; 2.2, *F*_6,102_ = 0.5003, *P* = 0.8069; 3.2, *F*_6,102_ = 1.4032, *P* = 0.2206; 5.5, *F*_6,102_ = 0.8332, *P* = 0.5470; repeated-measures ANOVA), Dunn’s multiple-comparisons test revealed significant differences between measurements taken at baseline and 1 day after noise exposure in response to 0.32 g/ms (*P* < 0.05) and 1.1 g/ms stimuli (*P* < 0.01).

### ABR

#### Threshold shift.

**Figure 5.**
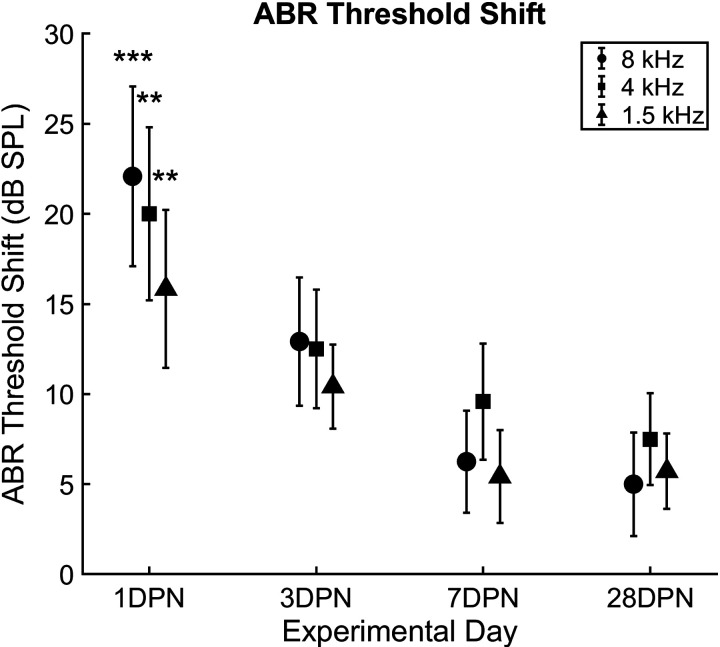
Auditory brainstem response (ABR) threshold shifts recovered over a 1-wk period following exposure to 110 dB sound pressure level (SPL) continuous noise. Threshold shifts were significantly different from baseline (*n* = 15) 1 day after noise exposure (*n* = 12) in response to all stimulus frequencies (8 kHz, circle; 4 kHz, square; 1.5 kHz, triangle) but were no longer significant 3 days after noise exposure (*n* = 12). Thresholds continued to recover to values near baseline 7 days after noise exposure (*n* = 12). Although ABR thresholds appeared elevated from baseline values when retested 28 days after noise exposure (*n* = 14), they were not statistically different from baseline and recovery was stable (**P < 0.01; ***P < 0.001). DPN, days postnoise; *n*, number of rats.

A significant threshold shift in ABR responses collected at 8 kHz (*F*_4,55_ = 6.2584, *P* < 0.001), 4 kHz (*F*_4,55_ = 5.0841, *P* < 0.01), and 1.5 kHz (*F*_4,55_ = 5.0331, *P* < 0.01) was observed (Fig. 5; one-way ANOVA). ABR thresholds were elevated 1 day after noise exposure (8 kHz, *P* < 0.001; 4 kHz, *P* = 0.001; 1.5 kHz, *P* < 0.01; Dunn’s multiple-comparisons test) before returning to values that appeared slightly elevated but were statistically the same as baseline.

#### Wave I amplitude.

**Figure 6.**
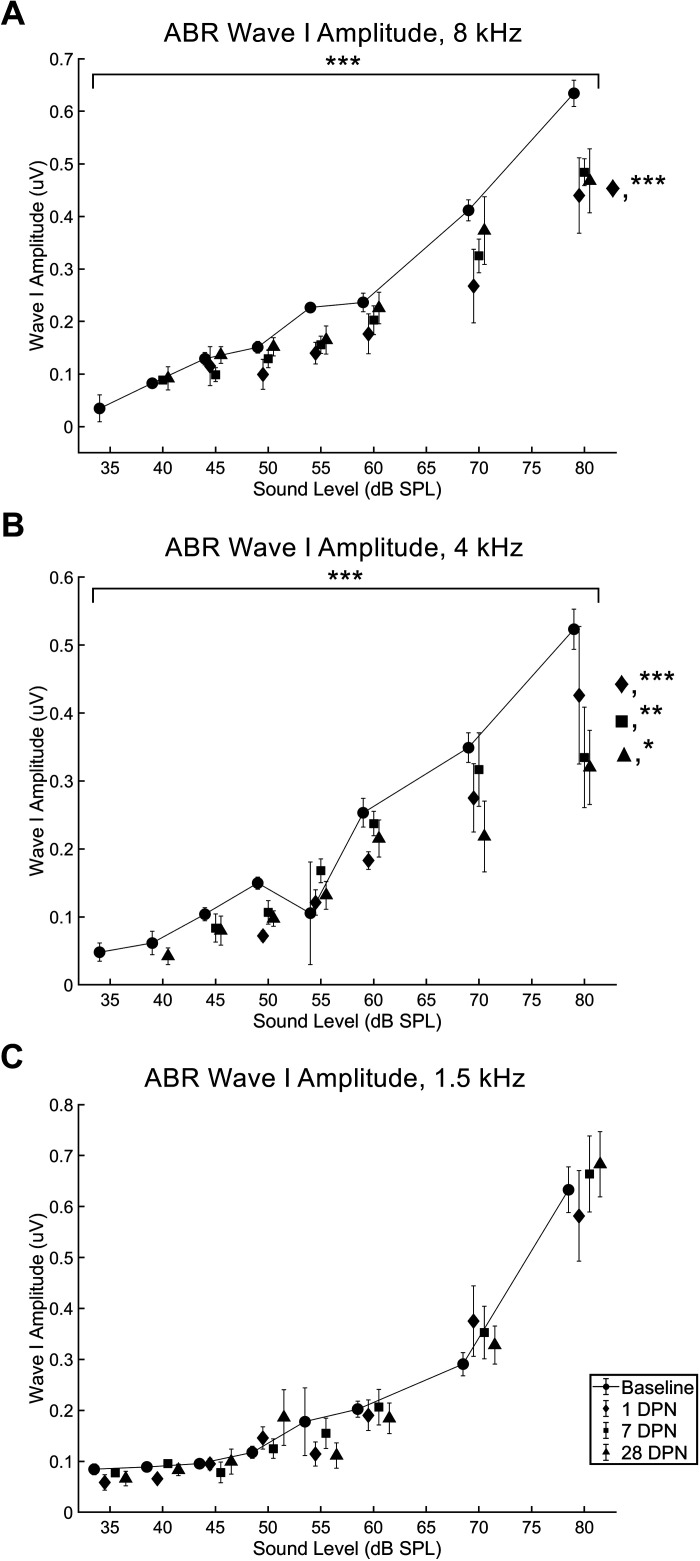
Auditory brainstem response (ABR) wave I amplitude was reduced following exposure to 110 dB sound pressure level (SPL) continuous noise and did not recover completely. ABR wave I amplitudes were significantly different from baseline (circles and solid line, *n* = 15) 1 day after noise exposure (diamonds, *n* = 11) in response to 8 kHz (*A*) and 4 kHz (*B*) stimulus frequencies but not in response to the 1.5 kHz stimulus (*C*). Although wave I amplitudes elicited by the 8 kHz pure tone stimulus were significantly different from baseline 1 day after noise exposure, they were not significantly different from baseline 3 (not shown) or 7 days (squares, *n* = 12) after noise exposure. This recovery was stable when retested 28 days after noise exposure (triangles, *n* = 13). Wave I amplitude in response to the 4 kHz stimulus was more severely impacted, with reduced amplitudes observed at all timepoints shown [*P < 0.05; **P < 0.01; ***P < 0.001, asterisks above bracket = group effect identified with ANOVA, asterisks to right of graph = significant findings 1 day (diamond), 7 days (square), and 28 days (triangle) after noise exposure identified with Dunn’s multiple-comparisons test]. DPN, days postnoise; *n*, number of rats.

After noise exposure, a significant effect of experimental day on wave I amplitude was observed in response to 8 kHz (*F*_4,370_ = 11.4295, *P* < 0.001; Fig. 6*A*) and 4 kHz (*F*_4,351_ = 5.8847, *P* < 0.001; Fig. 6*B*) but not 1.5 kHz (*F*_4,430 _= 1.5549, *P* = 0.1854; Fig. 6*C*) pure tones (two-way ANOVA). In response to 8 kHz pure tones, wave I amplitude was significantly reduced 1 day (*P* < 0.001) after noise exposure. In response to 4 kHz pure tones, wave I amplitude was significantly reduced 1 day (*P* < 0.001), 7 days (*P* < 0.01), and 28 days (*P* < 0.05; Dunn’s multiple-comparisons test) after noise exposure.

#### Wave I latency.

**Figure 7.**
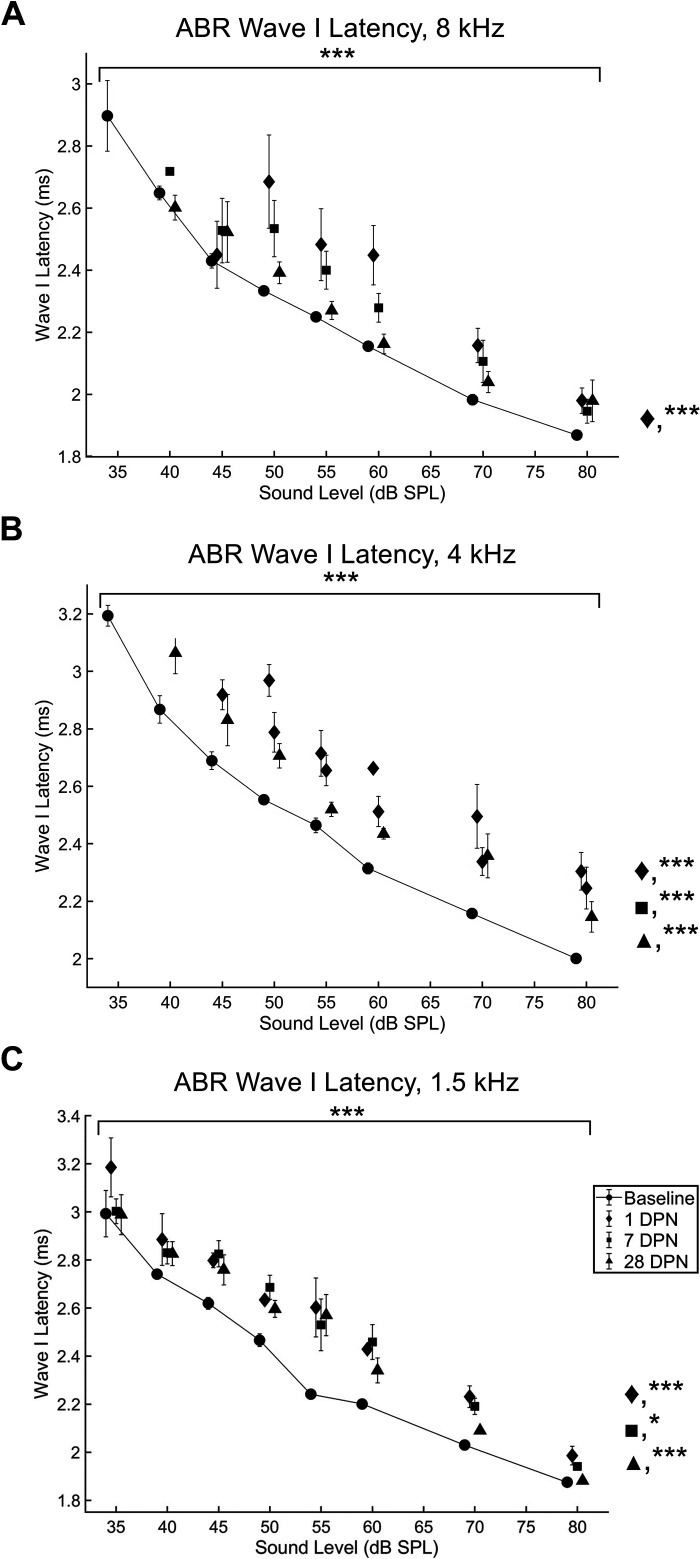
Auditory brainstem response (ABR) wave I latency was significantly prolonged following exposure to 110 dB sound pressure level (SPL) continuous noise and did not recover completely. ABR wave I latencies were significantly prolonged vs. baseline (circles and solid line, *n* = 15) 1 day after noise exposure (diamonds, *n* = 11) in response to all stimulus frequencies (8 kHz, *A*; 4 kHz, *B*; 1.5 kHz, *C*). Wave I latencies in response to the 8 kHz pure tone stimulus were significantly prolonged vs. baseline 1 and 3 days (not shown) after noise exposure. They were not significantly different from baseline 7 (squares, *n* = 12) or 28 days after noise exposure (triangles, *n* = 13). Wave I latencies in response to the 4 and 1.5 kHz stimulus were more severely impacted, with prolonged latencies observed at all timepoints [*P < 0.05; ***P < 0.001, asterisks above bracket = group effect identified with ANOVA, asterisks to right of graph = significant findings 1 day (diamond), 7 days (square), and 28 days (triangle) after noise exposure identified with Dunn’s multiple-comparisons test]. DPN, days postnoise; *n*, number of rats.

After noise exposure, a significant effect of experimental day on wave I latency was observed in response to 8 kHz (*F*_4,370_ = 32.2899, *P* < 0.001; Fig. 7*A*), 4 kHz (*F*_4,351_ = 48.6576, *P* < 0.001; Fig. 7*B*), and 1.5 kHz (*F*_4,425_ = 20.0994, *P* < 0.001; Fig. 7*C*) pure tones (two-way ANOVA). In response to 8 kHz pure tones, wave I latency was significantly elevated 1 day (*P* < 0.001) and 3 days (*P* < 0.01) after noise exposure. In response to 4 kHz pure tones, wave I latency was significantly elevated 1 day (*P* < 0.001), 3 days (*P* < 0.001), 7 days (*P* < 0.001), and 28 days (*P* < 0.001) after noise exposure. In response to 1.5 kHz pure tones, wave I latency was significantly elevated 1 day (*P* < 0.001), 3 days (*P* < 0.001), 7 days (*P* < 0.05), and 28 days (*P* < 0.001; Dunn’s multiple-comparisons test) after noise exposure.

## DISCUSSION

This study examined the effect of continuous noise exposure on ABR and VsEP responses in rats using a sound level 10 dB less than used in our previous studies (2, 18). Previous literature has linked noise-induced hearing loss with vestibular impairment in human (19, 20) and animal studies (2, 18; for review, see Ref. 1). The results demonstrate that 110 dB SPL exposure impairs VsEP responses, particularly those to small and moderate intensity head-jerk stimuli (0.32–3.2 g/ms) temporarily, with recovery occurring in the first postnoise week. Although previous work indicates that noise causes permanent NPVH (2), we now show that a 10 dB SPL reduction in sound intensity is sufficient to induce a transient NPVH. With the largest stimulus (5.5 g/ms), responses comparable with baseline measurements were elicited with the possible exception of reduced P2N2 amplitude that was significant (*P* < 0.01; Fig. 3*B*) 1 day after noise exposure. However, the P2N2 amplitude recovered to baseline by 3 days after noise exposure, similar to the recovery of P2N2 amplitudes to smaller stimuli. Although VsEP amplitude and latency are statistically the same as baseline within 3 days after noise exposure, responses to 0.32 g/ms head jerks were difficult to detect or not present (Fig. 1). Previous literature has shown either a lack of effect of noise on VsEP responses (21) or permanent effects as indicated by either partial or no recovery of VsEP waveforms following continuous noise exposure (2, 18).

A caveat to the data presented here is the possibility of auditory contamination of the VsEP. Auditory responses might be evoked either from bone-conducted vibration or from air-conducted sound produced by the shaker during head jerks. Some studies indicate that auditory contamination of the VsEP is small (12) but may affect VsEP waveform amplitude (21, 22). Recently, Jones and Lee (23) showed that forward masking to suppress auditory responses is of particular importance in mice for jerk durations less than 1 ms. The Jones and Lee study was based on one jerk intensity (2 g/ms) and did not determine whether auditory contamination could be significant in responses to larger jerk stimuli of short duration. Nevertheless, since we did not use forward masking, their finding suggests the possibility of auditory contamination of our VsEP responses. In particular, noise-induced attenuation of the VsEP may have been over-estimated if auditory contamination was greater in our baseline VsEP responses than in our postnoise VsEP responses. We do not believe this is the case. Before noise exposure, ABR and VsEP responses were robust; however, after noise exposure, ABR responses were significantly attenuated and failed to recover, whereas VsEP responses returned to baseline levels within 3 days in many animals and 1 wk in most. Based on prior work from Jones and Lee (23) and Jones et al. (17), the VsEP responses we report here reflect a dominant vestibular component. The middle (2.2 g/ms) stimulus that we used was similar in intensity to the stimulus used by Jones and Lee (23; +6 dB or 2 g/ms) and had a duration of 1.05 ms. According to Jones and Lee (23), a stimulus with these characteristics has minimal auditory contamination. Our data show that responses to 2.2 g/ms head jerks had transiently attenuated P1N1 amplitudes (Fig. 3*A*) and, unlike ABR responses, recovered by 3 days postnoise. Less intense jerks used in this study had durations greater than 1 ms; the 0.32 g/ms stimulus had a duration of 1.55 ms and the 1.1 g/ms stimulus had a duration of 1.35 ms. Responses to jerk stimuli greater than 1 ms exhibit little or no auditory contamination (23). The largest jerk stimuli that we used did have durations less than 1 ms; the 3.2 g/ms stimulus had a duration of 0.8 ms, and the 5.5 g/ms stimulus had a duration of 0.6 ms. Although responses to 3.2 g/ms head jerks had reduced P1N1 amplitudes 1 day after noise exposure, there was no significant change in responses elicited with the 5.5 g/ms stimulus at any timepoint (Fig. 3, *A* and *B*) despite significant attenuation of ABR responses in the same animals. Jones et al. (17) examined iso-acceleration stimuli up to 4.6 g/ms in the presence of auditory masking and reported robust vestibular responses despite short (0.5 ms) stimulus durations. These data show that strong vestibular responses can be elicited with intense jerk stimuli even when jerk duration is less than 1 ms. Furthermore, the recovery of baseline VsEP responses despite persistent attenuation of ABR responses implies that auditory contamination of our VsEP waveforms was relatively minor compared with the amplitude of the vestibular response. Future studies should address the interplay of jerk duration and jerk intensity to determine noise-induced threshold shifts in the absence of any auditory contamination.

Although ABR thresholds (Fig. 5) and 8 kHz ABR amplitudes and latencies recovered similarly to VsEPs, 4 kHz ABR amplitudes (Fig. 6) and 1.5 and 4 kHz latencies (Fig. 7) did not recover to baseline. ABR threshold shifts caused by 110 dB SPL noise exposure recovered quickly and to a much greater extent than in rats exposed to 120 dB SPL noise (Fig. 5; 2, 18); however, the amplitude and latency of the P1 waveform remained significantly different from baseline at the conclusion of the current study (Figs. 6 and 7). In previous studies (2, 18), ABR amplitude and latency was not reported due to lack of recovery. It appears that although ABR responses could be elicited following exposure to 110 dB SPL noise, the responses were abnormal.

Changes in hearing sensitivity after exposure to noise are well known. Temporary and permanent ABR threshold shifts have been reported in human and animal studies (for review, see Ref. 24). ABR threshold shifts typically recover within 7–10 days and may be related to middle ear damage, disruption of stereocilia bundles, or inflammatory processes in the cochlea and auditory nerve terminals. Even after recovery from temporary ABR threshold shifts, some patients experience difficulty understanding speech in complex auditory environments. Because these subjects have normal findings in routine audiometric tests, their deficit is characterized as “hidden hearing loss” (10, 25). Hidden hearing loss is associated with reductions in the number of cochlear nerve terminals and progressive loss of cochlear neurons in the absence of hair cell loss (10; for review, see Ref. 25). It would be interesting to compare VsEP recovery with testing of loss and recovery of balance or other vestibular functional metrics to see if there might be a vestibular equivalent of noise-induced hidden hearing loss.

Improvement of VsEP responses within 1 wk after 110 dB noise exposure (Fig. 2) suggests a temporary threshold shift in the stimulus intensity needed to evoke a detectable VsEP response. VsEP thresholds, although statistically the same as baseline measurements when measured 7 days after noise exposure (Fig. 2), remained elevated in 38% (8/21) of rats at the 7-day timepoint. At the conclusion of the study, 21% (4/19) of rats’ VsEP thresholds were elevated, suggesting that *1*) there is variability in the extent of recovery from 110 dB SPL noise exposure and *2*) VsEP responses following an apparent “temporary” deficit may not fully recover to baseline. Further studies will be required to determine the cellular basis for VsEP threshold shifts and whether they reflect reduced vestibular nerve activity caused by disruptions of transduction mechanisms (e.g., tip-link disruption) or synaptic transmission (e.g., synaptic loss), damage that may be cumulative with repeated noise exposures. In natural settings, it is unlikely that one would be exposed to high-intensity noise for hours at a time as was required to induce permanent changes in the rat’s VsEP. However, it is possible that multiple shorter exposures to 120 dB sound or multiple exposures to less intense sound (e.g., 110 dB) could cause cumulative damage to the vestibular periphery that might manifest as vestibular dysfunction (e.g., imbalance) over the time periods of several years as occurs with noise-induced hearing loss (NIHL). Additional studies are needed to determine the cellular mechanism(s) by which noise impacts VsEP responses and whether cellular damage in the vestibular periphery caused by repeated noise insults is cumulative over time.

## GRANTS

This work was funded by Department of Veterans Affairs (IK2RX003271, I01 RX003250-01A2) and National Institute on Deafness and Other Communication Disorders (R01 DC018003).

## DISCLOSURES

No conflicts of interest, financial or otherwise, are declared by the authors.

## AUTHOR CONTRIBUTIONS

C.E.S., D.S.B., R.A.A., and W.M.K. conceived and designed research; C.E.S., D.S.B., and W.M.K. performed experiments; C.E.S., D.S.B., R.A.A., and W.M.K. analyzed data; C.E.S., D.S.B., R.A.A., and W.M.K. interpreted results of experiments; C.E.S., R.A.A., and W.M.K. prepared figures; C.E.S. drafted manuscript; C.E.S., D.S.B., R.A.A., and W.M.K. edited and revised manuscript; C.E.S., D.S.B., R.A.A., and W.M.K. approved final version of manuscript.
